# Spatial Contrast Sensitivity to Polarization and Luminance in Octopus

**DOI:** 10.3389/fphys.2020.00379

**Published:** 2020-04-28

**Authors:** Luis Nahmad-Rohen, Misha Vorobyev

**Affiliations:** ^1^Leigh Marine Laboratory, Institute of Marine Science, University of Auckland, Auckland, New Zealand; ^2^Optometry and Vision Science, Faculty of Medical and Health Sciences, University of Auckland, Auckland, New Zealand

**Keywords:** octopus vision, octopus behavior, polarization vision, contrast sensitivity, polarization sensitivity, chromatic vision

## Abstract

While color vision is achieved by comparison of signals of photoreceptors tuned to different parts of light spectra, polarization vision is achieved by comparison of signals of photoreceptors tuned to different orientations of the electric field component of visible light. Therefore, it has been suggested that polarization vision is similar to color vision. In most animals that have color vision, the shape of luminance contrast sensitivity curve differs from the shape of chromatic contrast sensitivity curve. While luminance contrast sensitivity typically decreases at low spatial frequency due to lateral inhibition, chromatic contrast sensitivity generally remains high at low spatial frequency. To find out if the processing of polarization signals is similar to the processing of chromatic signals, we measured the polarization and luminance contrast sensitivity dependence in a color-blind animal with well-developed polarization vision, *Octopus tetricus*. We demonstrate that, in *Octopus tetricus*, both luminance and polarization contrast sensitivity decrease at low spatial frequency and peak at the same spatial frequency (0.3 cpd). These results suggest that, in octopus, polarization and luminance signals are processed via similar pathways.

## Introduction

Polarization sensitivity is widespread among invertebrates with rhabdomeric photoreceptors, such as arthropods and cephalopods, because rhabdomeric photoreceptors are inherently polarization sensitive by virtue of their microvillar structure (Moody and Parriss, 1960; Young, 1971). Polarization vision is particularly important to cephalopods, a group of animals that lack color vision, as it plays an important role in navigation (Shashar et al., 2002; Cartron et al., 2012), contrast enhancement (Shashar and Cronin, 1996; Shashar et al., 2002; Temple et al., 2012; Cartron et al., 2013b), detection of transparent prey (Shashar and Hanlon, 1998; Cartron et al., 2013a), and communication (Shashar and Cronin, 1996; Boal et al., 2004; Mäthger et al., 2009; Shashar et al., 2011). Furthermore, it seems that polarization contrast alone might be sufficient for both object detection and motion perception (Glantz and Schroeter, 2006; Pignatelli et al., 2011; Temple et al., 2012). It is possible that polarization vision could have an advantage over color vision in underwater environments, as the light spectrum is variable and long wavelengths (reds) are lost rapidly with depth, whereas the background polarization is relatively constant in angle and degree (Waterman, 1954; Marshall and Cronin, 2011), allowing for small changes in polarization to be easily detected in this almost noise-free environment (Cronin et al., 2003; Pignatelli et al., 2011).

It has been proposed that polarization vision is analogous to color vision in that it allows the viewer to divide a scene into regions based on the differences in polarization in the same way that color vision discriminates between chromaticities (Bernard and Wehner, 1977; Nilsson and Warrant, 1999; Cronin et al., 2003). Both polarization vision and color vision require a comparison between receptor signals: color vision is achieved through comparison of signals of photoreceptors tuned to different parts of the light spectrum (Vorobyev, 2007), whereas polarization vision is achieved through comparison of signals of photoreceptors tuned to different orientations of the electric field component of visible light.

If polarization vision were indeed analogous to color vision, a similar neural processing of both may be expected. One way to test this is through a contrast sensitivity function (CSF)—the dependence of sensitivity on the spatial frequency of a stimulus. The shape of the contrast sensitivity function depends on the neural processing of receptor signals (Barlow, 1961; Atick, 1992; Laughlin, 1994; Wandell, 1995). While the achromatic (luminance) contrast sensitivity function generally has a bell-like shape and decreases at both low and high spatial frequencies, chromatic sensitivity does not decrease at low spatial frequency and decreases at the high spatial frequency earlier than the achromatic contrast sensitivity function does (Cornsweet, 1970; Wandell, 1995). This difference between chromatic and achromatic contrast sensitivity has been demonstrated for humans, birds, fish, and insects (Mullen, 1985; Giurfa and Vorobyev, 1997; Lind and Kelber, 2011; Siebeck et al., 2014). The relationship between the shape of the contrast sensitivity and neural processing of visual signals is well-understood (Campbell and Robson, 1968; Cornsweet, 1970; Northmore and Dvorak, 1979; Rovamo et al., 1999; Pelli and Bex, 2013). While the decrease of sensitivity at low spatial frequency is a consequence of lateral inhibition, the lack of decrease of sensitivity at low spatial frequency is a consequence of spatial summation. A quantitative theory explaining the shape of contrast sensitivity has been developed for processing of luminance and chromatic information by human retinal ganglion cells (Rovamo et al., 1999).

In a previous paper we have demonstrated that the octopus luminance contrast sensitivity decreases at low spatial frequency (Nahmad-Rohen and Vorobyev, 2019). Here we compare luminance and polarization contrast sensitivity in *Octopus tetricus* (Gould, 1852). The lack of decrease of polarization sensitivity at low spatial frequency would be an indication of the similarity between processing of polarization in octopus and chromatic information in animals possessing color vision. On the other hand, the similarity of the shape of luminance and polarization sensitivity would indicate that luminance and polarization signals are conveyed via similar pathways.

Octopus photoreceptors are arranged in the form of parallel tubes packed into groups called rhabdomeres, the microvilli of which lie perpendicular to those of adjacent rhabdomeres and parallel to those of opposite ones. Such orthogonal arrangement provides polarization sensitivity as it strongly favors (and thus allows them to distinguish between) rays polarized at vertical and horizontal direction (Moody and Parriss, 1960, 1961; Young, 1971; Nilsson and Warrant, 1999). Octopuses are capable of discriminating between objects based on whether they show polarization contrast or not, and can detect variation in polarization patterns within a single object. Furthermore, they are able to identify objects through polarization cues alone, using the particular polarization patterns of each object to tell them apart (Shashar and Cronin, 1996; Cronin et al., 2003).

The polarization visual acuity of cephalopods has been measured through discrimination training (Shashar and Cronin, 1996), reflex method experiments (Temple et al., 2012), and optomotor tests (Cartron et al., 2013a). Discrimination tests have shown that octopuses can differentiate between polarization patterns with an *e*-vector variation of 20° (Shashar and Cronin, 1996). By presenting a polarized stimulus simulating an approaching predator and observing changes in body coloration as a response to it, Temple and colleagues reported that the minimum polarization angle difference detectable by octopus was 10° (Temple et al., 2012). Cephalopods have shown both optomotor and optokinetic responses in temporal resolution experiments designed specifically for polarization vision (Talbot and Marshall, 2010; Cartron et al., 2013a), and it was observed that, as with brightness, polarization sensitivity increases with age, albeit having a slower development (Cartron et al., 2013a).

However, thorough polarization contrast sensitivity measurements have not been made for octopuses. This may have been a very difficult (if not impossible) task to accomplish in the past, but it is now possible to do so by using a Liquid Crystal Display (LCD) screen. By removing the output polarizing filter, an image consisting of polarization patterns can be produced (Glantz and Schroeter, 2006; Foster et al., 2018). This technique has previously been used to present cuttlefish and squid with stimuli simulating either an approaching predator or a prey item and elicit the appropriate response (Pignatelli et al., 2011; Temple et al., 2012; Cartron et al., 2013b), as well as to present a looming stimulus to fiddler crabs (How et al., 2012). Another technique used for presenting butterflies with polarized stimuli includes using a digital light processing projector with a spinning linear polarizer (Stewart et al., 2017). Here we use a modified LCD screen to present octopuses with sinusoidal gratings to obtain a polarization contrast sensitivity function (PCSF).

Generally sensitivity depends on background. For example, in human observers, the sensitivity to changes of chromaticity decreases as the chromaticity of background increases, i.e., it is easier to detect small increase in chromaticity against achromatic backgrounds than against chromatic backgrounds due to the saturation of chromatic mechanisms (Krauskopf and Gegenfurtner, 1992). To find out if the ability of octopus to detect polarization depends on the polarization of background light, we test octopuses using unpolarized and 50% horizontally polarized background. The underwater light is horizontally polarized (Waterman, 1981; Marshall and Cronin, 2011), and the degree of polarization is typically below 50% and rarely exceeds 60%, even under exceptional conditions (Waterman, 1981; Marshall and Cronin, 2011). Therefore, the polarization of background light used in our experiments is within the range of variability of polarization of background light in the octopus habitat.

A novel method based on a reflex response was used to collect behavioral data from psychophysics experiments in a non-invasive way. This reflex response provides advantages over discrimination training methods, as it eliminates variables that can otherwise affect the test subject's choice, such as mood, confusion, and even handedness/preference for one side (Northmore and Yager, 1975; Byrne et al., 2002; Cartron et al., 2013a). Moreover, even when the conditioned response is well-established, curious animals can at times pick the wrong choice intentionally (Shashar and Cronin, 1996).

## Methods

### Animals and Housing

Ten octopuses (*Octopus tetricus*) (41–191 g) were captured with pot traps in Hauraki Gulf, New Zealand, and housed in individual tanks of 90 cm × 45 cm × 40 cm (L × W × H) with a 6 mm thick glass wall at the Leigh Marine Laboratory. Tanks had a constant flow of 200 μm filtered seawater from the Goat Island Marine Reserve, and were provided with an additional aeration system. Each tank was illuminated with overhanging fluorescent lights. Tanks were provided with an enriched, semi-natural environment (algae, rocks, and oyster shells), as well as a PVC pipe for octopuses to build their dens in. Animals were given live food (mussels and snails) 3–4 times/week. Individuals were given 2–3 days of acclimatization after capture before beginning experiments. Octopuses were kept for 2–3 months for experiments, after which they were released back into Hauraki Gulf.

All animal handling and experiments were carried out under approval of the University of Auckland Animal Ethics Committee (ref. 001761).

### Experimental Setup and Procedure

Vertical sinusoidal gratings of different spatial frequencies and varying contrast were used to measure the luminance and polarization contrast sensitivity of octopuses. The experimental procedure was based on that of Nahmad-Rohen and Vorobyev (2019).

Octopuses were presented with a stimulus consisting of vertically oriented sinusoidal gratings of five different spatial frequencies [12, 4, 1, 0.3, and 0.1 cycles per degree (cpd)] on an LCD screen to measure their contrast sensitivity to both luminance and polarization. The sinusoidal grating alternated between two positions by changing phase by 180° twice per second (temporal frequency of 2 Hz) to reduce habituation to the stimulus (Zeil, 2000). Depending on stimulus intensity, the octopus flicker-fusion frequency varies between 20 and 70 Hz (Hamasaki, 1968). We do not know which temporal frequency for the stimulus would be optimal for octopus vision. However, we empirically found that the frequency chosen (2 Hz) yields a reliable response. This alternating stimulus as opposed to a moving one was used to avoid confusion between contrast and motion sensitivity. Stimuli were created through MATLAB [version 7.12.0.635 (R2011a)] running Psychophysics Toolbox function interface (version 3.0.12 for Apple OS X, http://psychtoolbox.org).

The distance between the octopus and the tank edge was measured for each individual before beginning trials, and total distance was input into the script in order to generate the correct stimuli. Correction for the air-water interface was done by dividing the desired spatial frequency of the stimulus by the refractive index of seawater−1.34.

**Figure 1:**
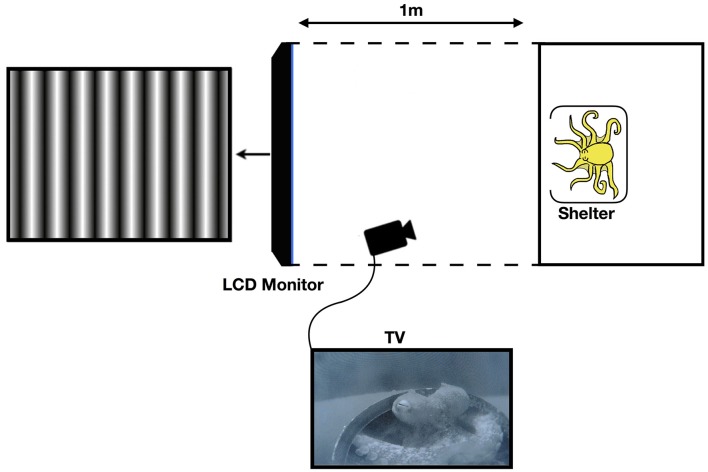
Diagram of the experimental setup (not to scale). The black corrugated plastic tunnel blocks any stimulus outside of the tank apart from the LCD monitor. Reprinted with permission from Nahmad-Rohen and Vorobyev (2019).

Black corrugated plastic was placed around the tank, as well as from the edge of the tank to the LCD monitor on the top, bottom, left, and right sides as a screen in order to restrict the octopus field of view of anything other than the monitor, so as to avoid any distractions or other visual stimuli. For this purpose, the aeration system was turned off during experiments. Furthermore, the lights above the tank were diffused, thus ensuring that the main light source was the LCD screen (see Figure 1).

The screen was located at 1 m from tank and always kept at conventional orientation. For luminance sensitivity measurements, the polarizer filter of the screen (DELL UltraSharp 1907FP, 19″) was, by manufacturer settings, oriented at 45° with respect to horizontal (0°) or vertical (90°) orientations. For polarization sensitivity measurements, the polarizer of the screen (DELL UltraSharp 1905FP, 19″), which was vertically oriented (90°) by manufacturer settings, was removed. The luminous intensity of the background against which the stimuli were presented was 86.7 for luminance sensitivity (50% gray), and 354.5 in the case of the screen with removed polarizer (the polarizing filter attenuates light emitted from the screen). The screen with the removed polarizer produces polarization patterns that do not have intensity contrast and hence are completely invisible to non-polarization sensitive animals. Thus, a maximum amplitude grating corresponded to perpendicular *e*-vector angles. By decreasing contrast, the degree of polarization difference in the grating is decreased. In the screen with the removed polarizer, pixel value 255 (white) corresponded to the vertical direction and pixel value 0 (black) corresponded to the horizontal direction.

To calibrate the modified LCD screen, a PR655 spectroradiometer (Photo Research Inc.) with affixed vertical (90°), horizontal (0°), and oblique (45° and 135°) polarizing filters was used to measure monitor output spectra at different pixel values (Figures A1-A,A2). The intensity values were obtained by multiplying spectra by the octopus spectral sensitivity (Brown and Brown, 1958) (Figure A1-B) and subsequent integration over wavelength. During calibration no change in intensity was observed at 45° and 135° for all pixel values, and measurements of the angle of polarization were constant across all intensities, meaning that changes in contrast or intensity correspond to changes in degree of polarization rather than to changes in *e*-vector orientation (see Appendix 1—Polarization Monitor Calibration). The pixel value at which the vertically and horizontally polarized light produced the same level of intensity determined the intensity corresponding to zero degree of polarization (see Figure A3). Gamma correction was introduced to ensure intensity linearity.

For the polarization contrast sensitivity measurements, two backgrounds were used: one with zero degree of polarization, the other with 50% polarization with a dominant horizontal *e*-vector orientation. The monitor settings used to achieve 50% polarization corresponded to the settings used for gray background in luminance experiments.

The uniform background (50% gray for unmodified LCD screen, unpolarized/50% polarization for modified screen) was shown for 1 h before the experiment began so that the octopuses became accustomed to it. Stimuli were presented for 2 s, shifting phase by 180° with a temporal frequency of 2 Hz. After stimulus presentation the programme remained idle, showing the uniform gray background, until given instruction to show the next one. A window of at least 20 s was used between stimulus presentations. To avoid habituation, frequencies were presented in random order. Each spatial frequency was presented a minimum of 30 times. To avoid habituation resulting from a stimulus with high Michelson contrast, the initial contrast for the procedure was 5%. Contrast was capped at 10% for the luminance experiment, and at 16% polarization for all frequencies except the highest one (12 cpd), which was capped at 28.6% polarization, for the polarization experiment. Thus, sensitivity values below 10 for luminance and 6.1 (or 3.5 for 12 cpd) for polarization cannot be reliably estimated. Minimum contrast, based on hardware limitations, was 0.7%.

Whether an octopus would see the stimulus or not was determined by whether a “fixation response” was shown—expansion of the chromatophores in and around the eye occurring as a result of accommodation, resulting in a momentary darkening around the pupil—(Turnbull, 2014; Nahmad-Rohen and Vorobyev, 2019). Because the fixation response is an uncontrolled reflex it does not require training, and preliminary tests in which the grating was shown at 100% contrast (see Nahmad-Rohen and Vorobyev, 2019—Supplementary Materials) showed that it is a reliable determinant of whether the grating is resolved by the subject.

The fixation response was monitored through a CCTV camera (Panasonic CCTV WV-BL200) placed outside the tank facing the octopus's eye. The camera provided live feed to a tv screen, making it possible to assess the presence or absence of a fixation response in real time. With each positive (fixation response present) or negative (no expansion of the chromatophores) response, contrast was decreased or increased, respectively, based on an adaptive staircase procedure with a Weibull approximation of the psychometric function (QUEST; Watson and Pelli, 1983). This procedure uses data from previous stimulus presentations to guide further testing. For each step, the adaptive staircase procedure uses the information from all previous trials to determine the contrast level of the next stimulus presentation and estimate the position of a threshold. This method allows us to establish a threshold reliably using only a limited number of trials (Watson and Pelli, 1983). The staircase was run until a contrast threshold was established, and a minimum of 30 presentations were done for each spatial frequency. Luminance sensitivity was defined as the inverse of the contrast threshold, and polarization sensitivity as the inverse of degree of polarization threshold.

It is important to note that, given the nature of the methods, we recorded attention threshold rather than minimal detectable contrast.

### Data Analysis

For each stimulus presentation, a Weibull psychometric function was applied to estimate the probability of stimulus detection:

(1) $$\begin{array}{l} \text{Ψ}\left(C;C_{t}\right)=\gamma +\left(1-\gamma -\delta \right)\left(1-exp\left(-{\left(\frac{C}{C_{t}}\right)}^{\beta }\right)\right), \end{array}$$

where γ is the false alarm rate (number of fixation responses in the absence of a stimulus), δ is the proportion of trials with blind responses (presentations in which the stimulus is resolvable by the individual—based on the contrast level—but still fails to respond), β is the slope of the psychometric function, *C* corresponds to the contrast presented, and *C*_*t*_ is the contrast threshold parameter searched for by the staircase procedure. We use the following values for false alarm rate and proportion of blind responses: γ = 0.0569, δ = 0.3058 (Nahmad-Rohen and Vorobyev, 2019). These values were obtained by presenting stimuli to two octopuses at 100% contrast. The proportion of blind responses was defined as the number of cases when the octopus did not respond to the 100% contrast grating divided by the total number of presentations. The false alarm rate was defined as the number of fixation responses when no stimulus was present divided by the total duration of the trial (Nahmad-Rohen and Vorobyev, 2019). The value for β (3) was based on suggestions from literature (Watson and Pelli, 1983).

Once the QUEST staircase procedure was finished, the threshold parameter *C*_*t*_ was calculated along with the error estimate for each spatial frequency. The local maximum of a likelihood function was then found while keeping parameters γ, δ, and β constant. The likelihood function *L* is:

(2) $$\begin{array}{l} L=\overset{n}{\underset{i=1}{\sum }}\left(y_{i}log\left(\text{Ψ}\left(C_{i},C_{t}\right)\right)+\left(1-y_{i}\right)log\left(1-\text{Ψ}\left(C_{i},C_{t}\right)\right)\right), \end{array}$$

where *n* is the total number of presentations for each given spatial frequency and *y* is the sequence of responses (0 if negative, 1 if positive) for each of the contrasts *C* at which the sinusoidal grating was presented. The value of the parameter *C*_*t*_ that maximized the likelihood function (i.e., for which the maximum value of the likelihood function was found) provides the contrast threshold. This was found using the “FindMaximum” function in Wolfram Mathematica^©^.

After obtaining the contrast threshold estimate, a parametric bootstrap procedure (Wichmann and Hill, 2001) was used to obtain error bars (**Figures 4**, **5**). This procedure was chosen because it takes into account the noise of the original data (Wichmann and Hill, 2001). In our case, the causes of noise can include factors such as changes in attention or distraction due to floating debris in the water. Assuming a binomial distribution of the data from the psychophysical experiments, and based on the probability of stimulus detection obtained from the Weibull psychometric function, a new set of data was created using a random number generator. In this virtual experiment, the stimulus was detected if the random number obtained was smaller than the probability of detection, and not detected if it was larger. Thus, a new sequence of responses *y* was obtained, in turn generating a new threshold *C*_*t*_ by maximizing the likelihood function with the virtual sequence of responses. For each spatial frequency, this procedure was repeated 10,000 times (obtaining different results of the virtual psychophysical experiment each time) to obtain a sequence of thresholds. The interquartile range of this threshold sequence is displayed as error bars in the contrast sensitivity graphs (**Figures 4**, **5**).

All data analysis was done in Wolfram Mathematica (version 11.3 for Mac OS X).

It is important to keep in mind that false alarms can occur as a result of different circumstances, such as floating debris in the water or neural noise. It is possible that the false alarm rate and proportion of blind responses vary between individuals.

### Relation Between Degree of Polarization and Contrast

We define the direction of polarization of the maximum signal (white, pixel value 255) as 0° and the direction of minimal signal (black, pixel value 0) as 90°. Let *I*_0_ and *I*_90_ be the intensities of light polarized at 0° and 90° orientations, respectively. Then, the degree of polarization is defined as:

(3) $$\begin{array}{l} P=\frac{I_{0}-I_{90}}{I_{0}+I_{90}}\;4 \end{array}$$

The degree of polarization, so defined, ranges from +1 to −1, with 0 corresponding to unpolarized light. Let $I_{0}^{0}$ and $I_{90}^{0}$ be the intensities corresponding to the background and $P^{0}=\;\frac{I_{0}^{0}-I_{90}^{0}}{I_{0}^{0}+I_{90}^{0}},$ then $\frac{2I_{0}^{0}}{I_{0}^{0}+I_{90}^{0}}=1+P^{0}$, $\frac{2I_{90}^{0}}{I_{0}^{0}+I_{90}^{0}}=1-P^{0}$. Note that, in the case of a screen with the removed polarizer, $I_{0}+I_{90}=I^{0}$ is constant. Therefore, when contrast *C* is presented against the background the light intensities are given by

(4) $$\begin{array}{l} I_{0}=I_{0}^{0}+CI_{0}^{0}\;=I^{0}\frac{\left(1+P^{0}\right)\left(1+C\right)}{2}, \end{array}$$

(5) $$\begin{array}{l} I_{90}=I_{90}^{0}-CI_{0}^{0}=I^{0}\left(1-\frac{\left(1+P^{0}\right)\left(1+C\right)}{2}\right), \end{array}$$

and substitution of Equation (5) into Equation (4) gives:

(6) $$\begin{array}{l} P=\frac{I_{0}^{0}-I_{90}^{0}+2CI_{0}^{0}}{I_{0}^{0}+I_{90}^{0}}=P_{0}+C\left(1+P^{0}\right). \end{array}$$

Therefore, the change of degree of polarization is given by:

(7) $$\begin{array}{l} P=C\left(1+P^{0}\right) \end{array}$$

## Results

**Figure 2:**
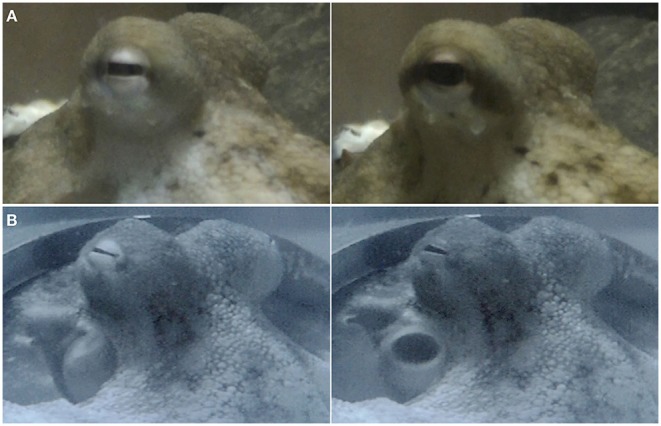
Two examples of octopus fixation reflex. (Left) Stimulus not present. (Right) Pupil expands along with chromatophores around the eye as a response to the appearance of the sinusoidal grating. The different features of the fixation response can be expressed at various degrees of intensity: **(A)** shows a strong pupil expansion and expression of the “eye bar,” whereas **(B)** shows a good example of the “dark eye ring.” **(B)** Reprinted with permission from Nahmad-Rohen and Vorobyev (2019).

**Figure 3:**
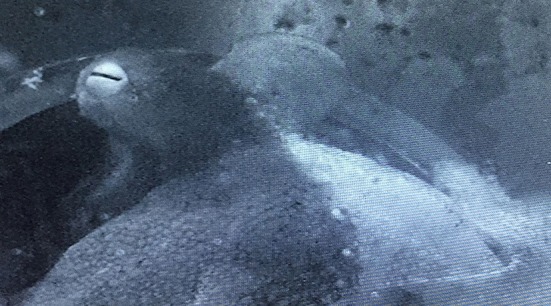
Octopus unilateral body pattern display.

The fixation response is comprised of three separate elements: expansion of the pupil, which can aid in estimating distances and which occurs in cephalopods when fighting or viewing food (Douglas et al., 2005); expression of the “dark eye ring” behavior, which can be seen in octopuses when disturbed by an object suddenly appearing or approaching the octopus (Borrelli et al., 2006); and expression of the “eye bar” (Packard and Sanders, 1971; Forsythe and Hanlon, 1985; Borrelli et al., 2006) (see Figure 2 and Supplementary Material Movie 1). Each one of these features can be displayed at various degrees of intensity, and they occur simultaneously. In some cases the dominant response is an expansion of the pupil, in others it is the darkening around the eye (dark eye ring and/or eye bar). However, it is difficult to specify in each case which one is dominant. During experiments it was observed that occasionally octopuses would not show a fixation response when a stimulus appeared on the screen, but would respond to it by displaying a unilateral body pattern—a conspicuous pattern in which one side of the body becomes dark while the other half remains pale (van Heukelem, 1966; Packard and Sanders, 1971; Forsythe and Hanlon, 1985; Borrelli et al., 2006) (see Figure 3). Contrary to the body pattern description by Forsythe and Hanlon (1985) and Packard and Sanders (1971), the dark side was not always the side facing the stimulus. Also, unlike Van Heukelem's description (van Heukelem, 1966), this color change was not frequent, and was only seen occasionally and clearly linked to the appearance of the sinusoidal grating. Most octopuses presented this pattern during the polarization experiments, and only one during the luminance experiments. These unilateral body pattern expressions were counted as “positive” responses for staircase procedure purposes.

**Figure 4:**
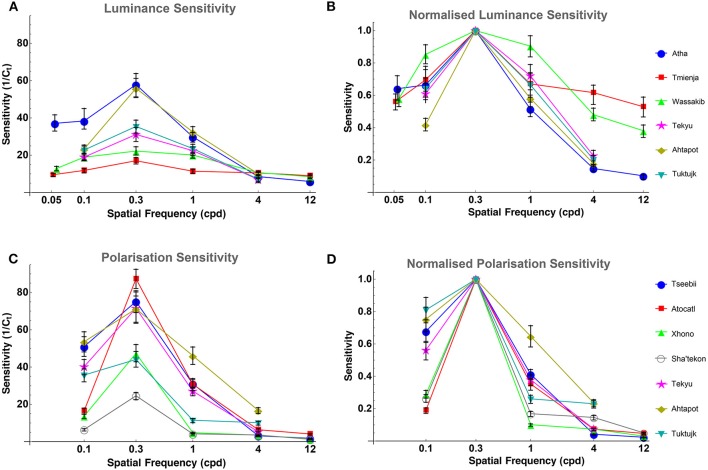
**(A)** Octopus LCSF. All octopuses show the same bell-like shape of the sensitivity curve, and all peak at 0.3 cpd. Sensitivity is defined as the inverse of the contrast threshold. The error bars indicate bootstrap interquartile range. **(B)** Octopus LCSF normalized to the peak sensitivity value of each curve (at 0.3 cpd) for easier comparison between them. **(C)** Octopus PCSF. All octopuses show the same bell-like shape of the polarization sensitivity curve as the ones from the luminance sensitivity experiment. Sensitivity is defined as the inverse of the contrast of the degree of polarization. The error bars indicate bootstrap interquartile range. **(D)** Octopus PCSF normalized to the peak sensitivity value of each curve (at 0.3 cpd) for easier comparison between them.

Luminance contrast sensitivity curves were obtained for three octopuses, and polarization contrast sensitivity curves were obtained for seven octopuses (three of which were the same as those for which luminance sensitivity curves were obtained). Three luminance contrast sensitivity thresholds from Nahmad-Rohen and Vorobyev (2019) are also presented here (octopuses 1–3: Atha, Tmienja, and Wassakib) Luminance contrast sensitivity peaked at 0.3 cpd and decreased at lower spatial frequencies in all six animals (see Figure 4). The mean contrast sensitivity at the maximum was 30.1 [contrast threshold = 3.32 ± 1.61% (mean ± SD)] and was in the range from 57.8 (contrast threshold = 1.73%) to 17 (contrast threshold = 5.87%) (see Table 1). By comparison, the best value for human contrast sensitivity is around 100 (contrast threshold = 1%) (Mullen, 1985).

**Table 1:** Contrast thresholds and sensitivity for luminance and polarization.

**Octopus**	**Contrast thresholds (%) and sensitivity (1/C**_****t****_**) at 0.3 cpd**	
	**Luminance**	**Polarization**
1. Atha	1.73 (57.8)	–
2. Tmienja	5.87 (17.0)	–
3. Wassakib	4.5 (22.2)	–
4. Tseebii*	–	1.33 (75.2)
5. Atocatl*	–	1.15 (87.3)
6. Xhono*	–	2.11 (47.3)
7. She'tekon*	–	4.07 (24.6)
8. Tekyu	3.21 (31.1)	1.44 (69.3)
9. Ahtapot	1.79 (55.9)	1.45 (69)
10. Tuktujk	2.82 (35.4)	2.34 (42.7)
Mean	3.32 (30.1)	1.96 (51)
SD (σ)	1.61 (17)	1.02 (21.8)

*Sensitivity is presented in brackets. For polarization sensitivity, octopuses marked with an asterisk (octopuses 4–7) were tested at 50% polarization background, while the others were tested at unpolarized background*.

Polarization contrast sensitivity also peaked at 0.3 cpd for six out of seven animals (see Figure 4). For one octopus (Tseebii) the polarization sensitivity at 1 cpd was higher (but not significantly) than that at 0.3 cpd. The staircase procedure was repeated for 1 cpd, yielding a much lower sensitivity (see Figure 4). Polarization contrast sensitivity was measured with unpolarized (3 animals: Tekyu, Ahtapot, Tuktujk) and 50% polarized (4 animals: Tseebii, Atocatl, Xhono, Sha'tekon) background. The degree of polarization sensitivities corresponding to the peak sensitivity (0.3 cpd) are equal to 59.2 [polarization threshold = 1.69 ± 0.5% (mean ± SD)] and 46.3 [polarization threshold = 2.16 ± 1.34% (mean ± SD)] for unpolarized and polarized backgrounds, respectively. The difference between sensitivities for polarized and unpolarized background was not significant (*p* = 0.84, *t*-test). The data from two groups were pooled together giving a mean polarization sensitivity of 51 [polarization threshold = 1.96 ± 1.02% (mean ± SD)]. The range of the polarization sensitivity was from 87.3 (polarization threshold = 1.15%) to 24.6 (polarization threshold = 4.07%) (see Table 1). The difference between the peak values of luminance and polarization thresholds was not statistically significant (*p* = 0.11, *t*-test).

**Figure 5:**
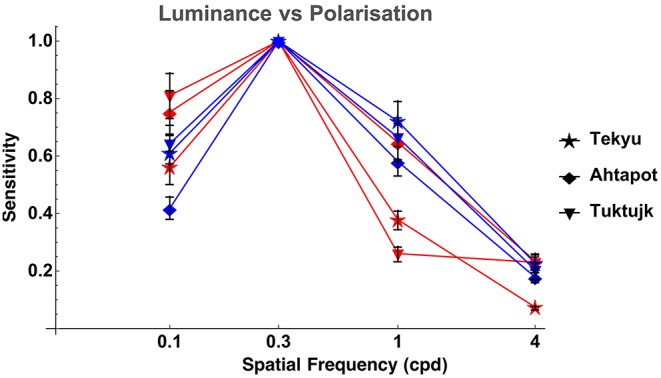
Comparison of LCSF (blue) and PCSF (red) of three octopuses (normalized to the peak sensitivity value of each curve at 0.3 cpd). The error bars indicate bootstrap interquartile range. It can be appreciated that the shapes of the sensitivity curves are quite similar, although the differences between them are inconsistent: polarization sensitivity is higher than luminance sensitivity for two octopuses (Ahtapot and Tuktujk) at low spatial frequency (0.1 cpd), and lower than luminance sensitivity at a higher spatial frequency (1 cpd) for two out of three octopuses (Tekyu and Tuktujk).

Comparison between luminance and polarization sensitivity curves from the three octopuses (Tekyu, Ahtapot, and Tuktujk) allowed us to investigate the difference between the luminance and polarization sensitivity in more details (Figure 5). The sensitivity maximum at 0.3 cpd was higher for polarization than for luminance for each of the three individuals (see Figure 5 and Table 1): 69.3, 69, and 42.7 for polarization vs. 31.1, 55.9, and 35.4 for luminance for Tekyu, Ahtapot, and Tuktujk, respectively. On the other hand, the difference in the shape was inconsistent. For two octopuses (Ahtapot and Tuktujk), polarization sensitivity was higher than luminance sensitivity at the lowest spatial frequency (0.1 cpd), and at a higher spatial frequency (1 cpd), polarization sensitivity was lower than luminance sensitivity for two octopuses (Tekyu and Tuktujk) (see Figure 5). To quantify the difference in the shape of contrast sensitivity curves, we normalized contrast sensitivity to its maximum at 0.3 cpd in all six luminance and seven polarization contrast sensitivity curves (see Figure 4). The normalized contrast sensitivities are characterized by the sensitivity at three frequencies (0.1, 1, and 3 cpd). The differences were assessed using a generalization of *t*-test to multidimensional data—the Hotteling *T*^2^-test followed by a Bonferroni *post-hoc* test. We detected a small but statistically significant difference (*p* = 0.026, *n*_1_ = 6, *n*_2_ = 7) between the shapes of the polarization and luminance contrast sensitivity (Figure 4). We found that the luminance sensitivity is significantly higher than the polarization sensitivity at 1 cpd (*p* = 0.007, Bonferroni *post-hoc* test), and no statistically significant differences for the other spatial frequencies.

## Discussion

We compared the contrast sensitivity to luminance and polarization in octopus and demonstrated that, in contrast to chromatic sensitivity, which generally does not decrease at low spatial frequency (Mullen, 1985; Giurfa et al., 1997; Lind and Kelber, 2011), octopus polarization sensitivity decreases at low spatial frequency. Therefore, we conclude that the processing of polarization signals in octopus is not analogous to the processing of chromatic signals in animals with color vision. Moreover, the shape of octopus polarization sensitivity is very similar to the shape of luminance sensitivity—both peak at the spatial frequency of 0.3 cpd. This indicates that luminance and polarization pathways are similar.

The difference in the processing of luminance and chromaticity can be explained in two different ways. (i) While luminance vision is achieved by summation of photoreceptor signals, chromatic vision is achieved by subtraction. This implies that chromatic vision suffers from the noise originating in photoreceptors to a higher degree than luminance vision. Therefore, chromatic vision has lower spatial resolution than luminance vision because spatial summation improves the signal-to noise ratio. Similar reasoning may explain the differences in the degree of lateral inhibition between luminance and chromatic vision. The lateral inhibition reduces the redundancy in the signal and hence improves the information transfer via channels with limited capacity (Barlow, 1961; Atick, 1992). However, when the signal-to-noise ratio is high it is beneficial to reduce or remove the lateral inhibition (Atick, 1992). (ii) Chromatic and achromatic vision are used for different purposes. Luminance vision is used for border detection, while chromatic vision is used for detecting the changes of material and identification of material properties (Rubin and Richards, 1982; Maximov, 2000). High spatial resolution and lateral inhibition facilitate border detection, but are not required for identification of material properties. Therefore, whereas chromatic vision is tuned to large visual angles, luminance (or achromatic) vision is tuned for detecting fine details in smaller visual angles (Giurfa et al., 1997; Lind and Kelber, 2011).

Because polarization vision is based on the comparison of two noisy signals, polarization vision may require larger spatial summation than luminance vision. Indeed, the only statistically significant difference between luminance and polarization sensitivity was a slightly lower contrast sensitivity for polarization at 1 cpd (see Figures 4, 5), which can be attributed to an increase of spatial summation for polarization vision compared to luminance vision. However, unlike chromatic vision, polarization vision cannot be used for identification of material properties because the perceived polarization depends on viewing conditions such as mutual positions of the observer and the object (Waterman, 1981; How and Marshall, 2014). Cephalopods use polarization vision for tasks that are similar in their nature to those associated with luminance vision. It has been demonstrated that cephalopods use polarization vision to detect transparent prey (Shashar and Hanlon, 1998; Cartron et al., 2013a) which usually consists of small, planktonic organisms with highly birefringent bodies (and therefore produce high polarization contrast) (Shashar and Hanlon, 1998; Johnsen et al., 2011). Polarization vision is also used by cephalopods for communication: depending on the individual's activity, polarization patterns in their bodies change (Boal et al., 2004; Chiou et al., 2007; Mäthger et al., 2009). These polarization patterns have fine details (Boal et al., 2004; Chiou et al., 2007; Mäthger et al., 2009). Hence, the similarity between luminance and polarization contrast sensitivity and the presence of lateral inhibition in polarization vision is consistent with the similarity of tasks of luminance and polarization vision. However, it cannot be excluded that polarization vision can be also used for tasks that do not require high spatial resolution, such as polarization-based navigation (Shashar et al., 2002; Cartron et al., 2012). While it is well-established that, in terrestrial habitats, insects use polarization vision for navigation (Labhart, 2016), the utility of polarization cues for navigation are not well-understood. It has been argued that polarization-based navigation underwater is restricted to very shallow waters, and could be limited to shore detection (Shashar et al., 2011).

Due to the limitations of the methods, polarization sensitivity was measured at a higher light intensity than that used to measure luminance sensitivity. This can contribute to a possible difference in the maximal sensitivities to luminance and polarization (note that the difference in the maximal sensitivity between luminance and polarization was not statistically significant). However, the differences in the light levels at which the experiments were performed cannot explain the decrease of polarization sensitivity at 1 cpd because with the increased light level the sensitivity to high frequency should increase due to decrease of spatial summation (Atick, 1992). We performed experiments polarized and 50% polarized background and did not detect any significant difference. Because natural background polarization very rarely exceeds 50% (Waterman, 1981; Marshall and Cronin, 2011), we conclude that polarization sensitivity is unlikely to saturate in natural conditions.

Cartron et al. (2013a) noted that, in insects “polarized and unpolarized information are coded differently and are processed by different type of neurons in the optic lobe,” and provide the hypothesis that “In cephalopods […] polarization is not a simple modulation of luminance information, but rather that it is processed as a distinct channel of visual information.” Our results do not confirm this hypothesis and indicate that the processing of polarization and luminance signals in octopus are largely similar.

## Data Availability Statement

All datasets generated for this study are included in the article/Supplementary Material.

## Ethics Statement

The animal study was reviewed and approved by University of Auckland Animal Ethics Committee (ref. 001761).

## Author Contributions

MV designed the experiment. LN-R performed the experiments. Both authors analyzed the data and wrote the manuscript.

## Conflict of Interest

The authors declare that the research was conducted in the absence of any commercial or financial relationships that could be construed as a potential conflict of interest.

## Supplementary Material

Supplementary Material Movie 1 Octopus fixation reflex.

## Abbreviations

LCSF: luminance contrast sensitivity function
PCSF: polarization contrast sensitivity function.
