# Supplementary material for: Spatial Contrast Sensitivity to Polarization and Luminance in Octopus
Source: Front Physiol. 2020 Apr 28;11:379. doi: 10.3389/fphys.2020.00379 (PMC7212343; doi:10.3389/fphys.2020.00379)
Supplement: Supplementary file 3 [file Data_Sheet_2.pdf]

Appendix

Polarisation Monitor Calibration

Calibration of the polarisation monitor (monitor with removed polariser) was done in order to **(i)** determine how degree of polarisation, P, and polarisation angle (*e*-vector) changed as a function of intensity (pixel value), **(ii)** accurately control the level of polarisation contrast presented to the octopus in the sinusoidal grating stimulus, and **(iii)** determine the position of the neutral point – the intensity at which the degree of polarisation is equal to zero.

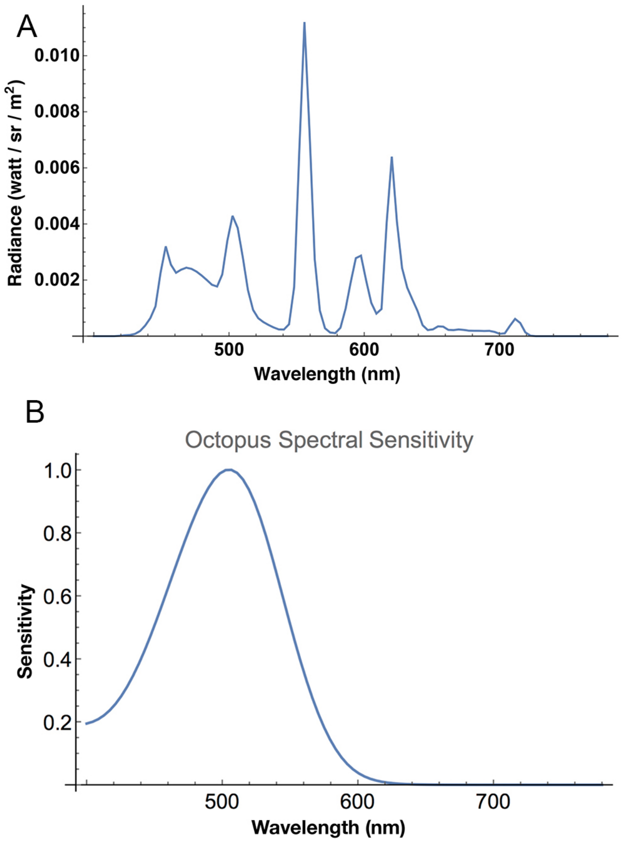

Figure A1: **(A)** Example of spectral composition of polarisation monitor during calibration. **(B)** Octopus spectral sensitivity.

A PR-655 SpectraScan® spectrometer (Photo Research Inc.) (sampling range: 380-780nm, sampling rate: 4nm) with affixed polarising filters in four different orientations – vertical (90˚), horizontal (0˚), and oblique (45˚ & 135˚) – was used to measure monitor output spectra at different pixel values (0-255 at increment intervals of 10) for each orientation (see Figure A1-A). The pixel values for the three channels (RGB) were kept at the same level, i.e. the input to the monitor corresponded to different shades of grey. It is important to note that pixel values of 0 & 255 correspond to black and white respectively, for the vertical orientation of the polarising filter (original orientation of the monitor's filter), with intermediate pixel values corresponding to different levels of grey; this is the inverse for the horizontal orientation, with pixel values of 0 & 255 corresponding to bright and dim respectively.

The degree of polarisation and angle of polarisation were found using Stokes parameters (Berry et al., 1977; Hecht, 2002)

$$S_0 = I \qquad \text{A1a}$$

$$S_1 = I_{0^\circ} - I_{90^\circ} \qquad \text{A1b}$$

$$S_2 = I_{45^\circ} - I_{135^\circ} \qquad \text{A1c}$$

where I is the total light intensity, I_α is the intensity of light transmitted at angle α. The angle of polarisation (or *e*-vector orientation) can be calculated as

$$\Theta = \frac{1}{2} arctan(\frac{S_2}{S_1}) \qquad \text{A2}$$

and degree of polarisation as

$$P = \frac{\sqrt{S_1^2 + S_2^2}}{S_0} \quad . \qquad \text{A3}$$

Eq. 3 is equivalent to the definition of degree of polarisation given by Eq. 4 if I_{0° and I_{90° correspond to maximum and minimum

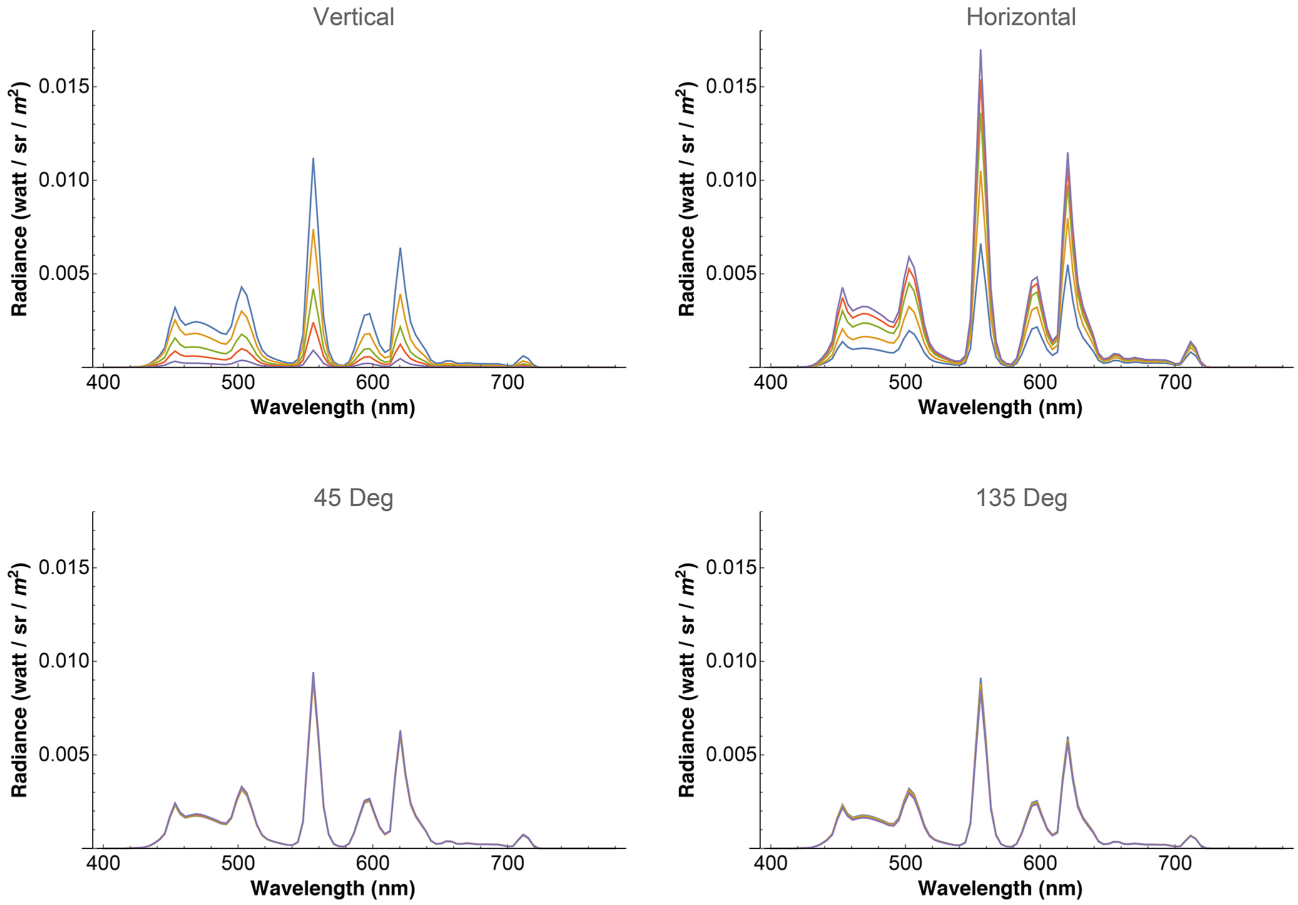

Figure A2: Results of calibration for the monitor with the front polarising filter removed: Spectral composition at 5 different pixel values (255, 200, 140, 100, & 60) for vertical, horizontal, 45˚, & 135˚ filter orientations. Note that for 45˚ & 135˚ there is no change in intensity with pixel value.

intensities and $I_{45^\circ} = I_{135^\circ}$. Note that the measurement of light intensity at four orientations of polariser $(I_{0^\circ}, I_{90^\circ}, I_{45^\circ}, I_{135^\circ})$ is redundant and the angle and degree of polarisation can be found using only three orientations of polariser.

Figure A2 shows examples of the light spectra at different pixel values for all filter orientations. For vertical and horizontal orientations, intensity changed with pixel value, whereas spectral composition remained practically constant. Furthermore, overall intensity measured through the horizontally oriented filter was higher than the corresponding one measured through the vertically oriented filter for the corresponding pixel values, e.g. pixel value 0 at horizontal orientation of the filter had higher intensity than pixel value 255 at vertical orientation. At 45˚ and 135˚ orientations, output intensity remained constant and equal to each other for all pixel values. Therefore the angle of polarisation was either 0° or 90° (see Eq. A2). This means that intermediate levels of grey correspond to changes in degree of polarisation, while the *e*-vector orientation remains constant.

Because there is no change in angle of polarisation, Eq. A3 can be simplified to obtain the degree of linear polarisation (see section 2.5 - Methods: Relation between Degree of Polarisation and Contrast, Eq. 4). The degree of polarisation was found to be extremely high (99.5%) at pixel value 0, decrease to 0% at pixel

value 205, and increase again to 35% at pixel value 255 (see Figure A3).

For each of the pixel values, the intensity for octopus was calculated taking into account the octopus spectral sensitivity (Brown & Brown, 1958). The spectral sensitivity was modelled using Govardovskii templates (Govardovskii et al., 2000) assuming that the visual pigment peaks at 475 nm (see Figure A1-B). The intensity was calculated as

$$I = \int J(\lambda)S(\lambda)d\lambda, \qquad \text{A4}$$

where $J(\lambda)$ denotes the light spectrum and $S(\lambda)$ is the spectral sensitivity.

In order to use an unpolarised background over which the stimuli were presented it was necessary to determine the position of the neutral point. As expected, it was observed that vertical and horizontal intensities show inverse behaviours: vertical intensity increased with pixel values, whereas horizontal intensity decreased. Where these two intersected (i.e. the pixel value at which the vertical and horizontal intensities were equal) determined the position of the neutral point – the intensity at which the degree of polarisation is equal to zero (see Figure A3). Note that it coincides with the point at which degree of linear polarisation equals zero in Figure A3.

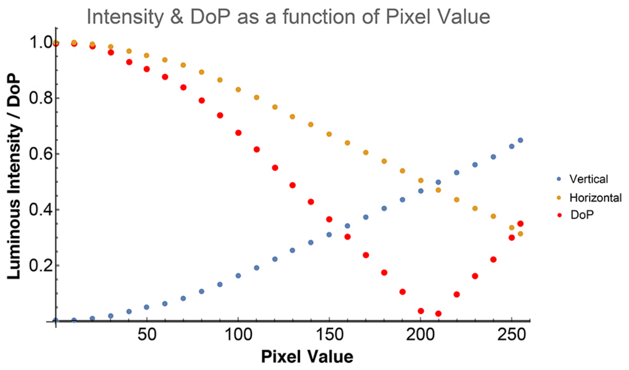

Figure A3: Degree of linear polarisation and vertical and horizontal luminous intensities (normalised to maximum horizontal intensity) as functions of pixel value. It can be appreciated that horizontal output intensity reached higher levels than vertical output intensity. The intersection point of these two curves corresponds to the intensity (and pixel value) at which the degree of polarisation is equal to zero. Note that the point at which DoP reaches zero and the intersection between vertical & horizontal intensities are the same.
